# The Mechanism of Ferroptosis and Blood–Brain Barrier Damage in Cerebrovascular Diseases

**DOI:** 10.3390/biomedicines14030604

**Published:** 2026-03-09

**Authors:** Jiaxin Guo, Chengye Yao

**Affiliations:** Department of Neurology, First Clinical College, Tongji Medical College, Huazhong University of Science and Technology, Wuhan 430022, China

**Keywords:** ferroptosis, blood–brain barrier, cerebrovascular diseases, ischemic stroke, hemorrhagic stroke, neuroprotection

## Abstract

The blood–brain barrier (BBB) is a vital protective structure that prevents harmful substances in the blood from entering the central nervous system while maintaining homeostasis. Its dysfunction can lead to significant pathological changes and contribute to various neurological disorders, such as stroke and neurodegenerative diseases. BBB damage of varying degrees is observed in nearly all cerebrovascular diseases, yet the specific mechanisms remain largely unclear. Growing evidence indicates that ferroptosis—an iron-dependent, lipid peroxidation-driven form of regulated cell death—is a major pathway contributing to BBB disruption. Ferroptosis affects multiple key brain cell types, including endothelial cells, glial cells, pericytes, and neurons, potentially leading to BBB dysfunction in cerebrovascular diseases. This article explores the role of ferroptosis in different types of cerebrovascular diseases and its effects on various cells. It covers the latest research in this area and highlights the potential benefits of targeting ferroptosis, including protecting the blood–brain barrier, reducing neuroinflammation, and protecting neurons.

## 1. Introduction

Cerebrovascular diseases (CVDs) are a group of disorders caused by structural or functional abnormalities in cerebral blood vessels, leading to impaired cerebral blood flow and subsequent ischemic, hypoxic, or hemorrhagic damage to brain tissues. The main subtypes include ischemic stroke and hemorrhagic stroke, both of which can cause irreversible brain damage and neurological deficits, leading to high mortality and disability rates. Despite being a global health threat, treatment options remain limited. Thrombolytic therapy is the only reperfusion treatment for ischemic stroke approved by the U.S. Food and Drug Administration. However, due to its narrow treatment window, the actual usage rate of this therapy is extremely low. Hemorrhagic stroke treatment primarily relies on minimally invasive surgery, which can cause secondary damage like brain edema and limited post-operative recovery [1]. Neuroprotective agents are always a major focus, but the complex pathophysiology involving endothelial dysfunction, inflammation, oxidative stress, and neuronal injury complicates progress. The introduction of the neurovascular unit (NVU) concept has shifted the focus of neuroprotection from merely protecting neurons to also safeguarding the function of various cells and the intercellular signaling pathways within the entire functional NVU [2].

Ferroptosis, a form of iron-dependent cell death driven by lipid peroxidation, is characterized by the accumulation of reactive oxygen species (ROS) and lipid peroxides, ultimately leading to cell membrane rupture [3]. The importance of ferroptosis in CVDs may stem from the unique physiological properties of the brain. The brain is composed of approximately 50% lipids, particularly polyunsaturated fatty acids (PUFAs) found abundantly in neuronal membranes, making it more susceptible to lipid peroxidation [4]. Furthermore, accounting for 20% of systemic oxygen consumption, the brain maintains high metabolic demands requiring sustained ATP production to preserve neuronal homeostasis, thereby generating excessive mitochondrial-derived reactive species under ischemic conditions. Research has shown that during ischemic stroke, disrupted iron homeostasis and oxidative stress synergistically activate ferroptotic cascades, compromising the blood–brain barrier’s integrity and exacerbating inflammatory responses [5]. In hemorrhagic stroke, the release of hemoglobin and heme from ruptured red blood cells increases local iron levels, triggering oxidative stress and ferroptosis, which accelerates disease progression and worsens prognosis [6,7]. Recent studies have also found that ferroptosis not only involves multiple pathological processes but also affects various brain cell types, including endothelial cells (ECs), glial cells, pericytes, and neurons, forming a complex pathological network. This may provide a broader range of intervention strategies compared to traditional treatments.

In conclusion, a deeper understanding of the specific mechanisms of ferroptosis in CVDs and its pathological effects on different brain cell types not only offers new insights into stroke pathophysiology but also presents novel therapeutic approaches. We highlight the therapeutic potential of targeting ferroptosis as a multi-target strategy to reduce secondary neuronal damage and improve neurological outcomes.

## 2. Ferroptosis

**Figure 1:**
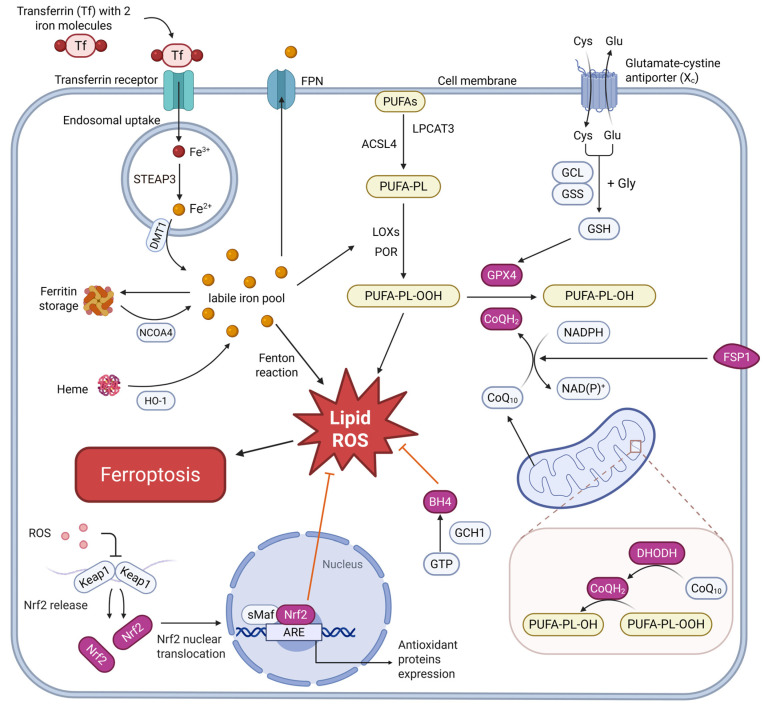
Molecular mechanisms of ferroptosis. Ferroptosis is a regulated form of cell death characterized by iron-dependent lipid peroxidation and subsequent plasma membrane rupture. It can occur through two main pathways: (1) iron ion accumulation and (2) lipid peroxidation accumulation. Various enzymes and molecules, including LOXs, GPX4, CoQ_10_, and Nrf2, play crucial roles in regulating ferroptosis and maintaining cellular homeostasis. Abbreviations: ACSL4, acyl-CoA synthetase long-chain family member 4; ARE, antioxidant response element; BH4, tetrahydrobiopterin; CoQ_10_, coenzyme Q_10_ (ubiquinone); CoQH_2_, reduced coenzyme Q (ubiquinol); Cys, cysteine; DHODH, dihydroorotate dehydrogenase; DMT1, divalent metal transporter 1; Fe^2+^, ferrous iron; Fe^3+^, ferric iron; FPN, ferroportin; FSP1, ferroptosis suppressor protein 1; GCH1, GTP cyclohydrolase 1; GCL, glutamate-cysteine ligase; Glu, glutamate; Gly, glycine; GPX4, glutathione peroxidase 4; GSH, glutathione (reduced form); GSS, glutathione synthetase; HO-1, heme oxygenase-1; Keap1, Kelch-like ECH-associated protein 1; LOXs, lipoxygenases; LPCAT3, lysophosphatidylcholine acyltransferase 3; NADPH, nicotinamide adenine dinucleotide phosphate (reduced form); NAD(P)^+^, oxidized nicotinamide adenine dinucleotide (phosphate); NCOA4, nuclear receptor coactivator 4; Nrf2, nuclear factor erythroid 2-related factor 2; POR, cytochrome P450 oxidoreductase; PUFAs, polyunsaturated fatty acids; PUFA-PL, PUFA-containing phospholipid; PUFA-PL-OH, reduced PUFA-phospholipid alcohol; PUFA-PL-OOH, PUFA-phospholipid hydroperoxide; ROS, reactive oxygen species; sMaf, small Maf protein; STEAP3, six-transmembrane epithelial antigen of prostate 3; System Xc^−^, cystine/glutamate antiporter; Tf, transferrin. Created in BioRender. Guo, J. (2026) https://BioRender.com/5ry5w8r (accessed on 8 February 2026).

Ferroptosis is an iron-dependent, regulated form of cell death characterized by lipid peroxidation and oxidative stress. It is initiated when cellular antioxidants fail to prevent the accumulation of lipid peroxides, leading to membrane rupture and cell death [3]. In the context of CVDs, ferroptosis contributes significantly to the dysfunction of the NVU, particularly in ECs, pericytes, astrocytes, and microglia. To better illustrate the complex network of ferroptosis regulation, including lipid peroxidation, iron metabolism, and antioxidant systems, a comprehensive diagram is provided below (Figure 1). This diagram visually highlights the key molecular events of ferroptosis, emphasizing the central role of lipid ROS, iron overload, and the imbalance between oxidative and antioxidant systems.

### 2.1. Accumulation of Iron

Iron dyshomeostasis is a central driver of ferroptotic cell death across multiple cell types within the NVU. At the level of the BBB, ECs internalize circulating transferrin-bound Fe^3+^ via transferrin receptor 1 (TfR1)–mediated endocytosis. Within endosomes, Fe^3+^ is reduced to Fe^2+^ by six-transmembrane epithelial antigen of the prostate (STEAP) family ferrireductases, such as STEAP3, and Fe^2+^ is subsequently transported into the cytosol through divalent metal transporter 1 (DMT1), thereby contributing to the endothelial labile iron pool. Cytosolic Fe^2+^ can then be exported across the abluminal membrane via ferroportin and re-oxidized by ferroxidases including ceruloplasmin and hephaestin, allowing Fe^3+^ to rebind transferrin in the interstitial fluid and be taken up by neurons and glial cells [5,8]. In addition, intracellular iron is further controlled through ferritin storage and nuclear receptor coactivator 4 (NCOA4)-dependent ferritinophagy, as well as heme oxygenase-1 (HO-1)-mediated heme degradation, which release ferrous iron under stress conditions [9,10]. Excessive activation of these iron-releasing pathways in acute or chronic disease leads to accumulation of labile Fe^2+^, enhanced Fenton chemistry, hydroxyl radical generation and lipid peroxidation [3].

Importantly, the canonical TfR–STEAP–DMT1–mediated transferrin cycle that generates a cytosolic labile iron pool is well established in ECs and neurons, supporting trans-BBB iron transport and neuronal iron supply [8,11]. By contrast, astrocytes, microglia and pericytes appear to rely mainly on alternative iron-handling mechanisms, including ferritin-based storage, uptake of non–transferrin-bound iron, and ferroxidase-mediated iron export via ceruloplasmin and hephaestin. In particular, astrocytes and pericytes provide substantial ferrous oxidase activity, promoting the re-oxidation of Fe^2+^ to Fe^3+^ and facilitating iron export and redistribution to neighboring cells, thereby buffering local iron fluctuations at the vascular–parenchymal interface [8].

### 2.2. Accumulation of Reactive Oxygen Species

ROS are a key feature of ferroptosis, driven by mitochondrial dysfunction, NADPH oxidase (NOX) activity, and LOX-mediated peroxidation of PUFAs [3,12,13]. Iron, abundant in the brain, drives ROS generation through Fenton reactions, lipid autoxidation, and LOX activation, leading to lipid peroxidation [14]. These oxidative processes induce multi-cellular damage within the central nervous system.

### 2.3. Lipid Peroxidation

Ferroptosis is driven by the peroxidation of phospholipids containing PUFAs in the cell membrane, mediated by Fe^2+^ [14]. This process involves both enzymatic and non-enzymatic pathways: Fe^2+^ generates free radicals through the Fenton reaction, initiating non-enzymatic chain reactions. In enzymatic reactions, lipoxygenases catalyze the oxidation of PUFAs, forming lipid hydroperoxides [3,4]. The accumulated lipid hydroperoxides further decompose, producing reactive aldehydes like 4-hydroxynonenal and malondialdehyde, which damage the cell membrane and exacerbate oxidative stress. Additionally, acyl-CoA synthetase long-chain family member 4 (ACSL4) and lysophosphatidylcholine acyltransferase 3 enhance the accumulation of PUFAs in the cell membrane, increasing its susceptibility to damage [15,16].

### 2.4. Antioxidant Defense Mechanisms

#### 2.4.1. GPX4-Dependent Antioxidant Network: System Xc^−^/GSH/GPX4 and Nrf2

The canonical antioxidant defense against ferroptosis is mediated by the System Xc^−^-GSH-GPX4 axis. System Xc^−^, a cystine/glutamate antiporter composed of solute carrier family 7 member 11 (SLC7A11, also known as xCT) and solute carrier family 3 member 2, imports extracellular cystine, which is rapidly reduced to cysteine and thereby supports glutathione (GSH) biosynthesis [17]. GSH prevents iron-dependent oxidation partly by binding Fe^2+^ and, more importantly, by serving as an essential cofactor for glutathione peroxidase 4 (GPX4), which reduces lipid hydroperoxides to non-toxic lipid alcohols and thus maintains membrane redox homeostasis [18]. Inhibition of this axis—through blockade of System Xc^−^, depletion of GSH, or direct inactivation of GPX4—decreases intracellular GSH levels, inactivates GPX4, and triggers uncontrolled lipid peroxidation, eventually leading to ferroptotic cell dysfunction and death [18].

At the transcriptional level, this pathway is tightly regulated by nuclear factor erythroid 2-related factor 2 (Nrf2). Under basal conditions, Nrf2 is sequestered in the cytoplasm by Kelch-like ECH-associated protein 1 and targeted for proteasomal degradation. Upon oxidative stress, Nrf2 dissociates from Kelch-like ECH-associated protein 1, translocates to the nucleus, and binds to antioxidant response elements (AREs) to activate the transcription of a broad set of cytoprotective genes [19]. Among these targets, Nrf2 upregulates SLC7A11 and key enzymes involved in GSH synthesis, as well as GPX4, thereby strengthening the System Xc^−^-GSH-GPX4 axis [19]. Silencing or inhibition of Nrf2 makes cells more sensitive to ROS accumulation and accelerates ferroptosis [20]. Thus, the Nrf2-ARE pathway functions as an upstream regulator that boosts the GPX4-dependent antioxidant network and confers resistance to ferroptotic injury.

#### 2.4.2. GPX4-Independent CoQ/BH4 Systems: FSP1, DHODH and GCH1

In addition to the canonical System Xc^−^-GSH-GPX4 axis, cells possess several GPX4-independent defense systems that converge on coenzyme Q10 (CoQ_10_/CoQH_2_) and tetrahydrobiopterin (BH4). Together, these pathways form a spatially and functionally compartmentalized antioxidant network that constrains lipid peroxidation and modulates susceptibility to ferroptosis.

Among these systems, the Ferroptosis suppressor protein 1 (FSP1)-CoQ_10_-NAD(P)H axis constitutes a major extramitochondrial line of defense. FSP1 is an NADPH-dependent oxidoreductase that localizes to the plasma membrane and lipid droplets in an N-myristoylation-dependent manner [21]. FSP1 utilizes NAD(P)H to reduce oxidized CoQ_10_ (ubiquinone) to its active, reduced form ubiquinol (CoQH_2_), which acts as a lipid radical-trapping antioxidant and terminates lipid peroxidation chain reactions independently of GPX4 [21,22]. More recent studies have shown that FSP1 also participates in the vitamin K redox cycle and can recruit ESCRT-III complexes to repair oxidatively damaged plasma membranes, further contributing to ferroptosis resistance [23,24]. CoQ_10_ is able to reach the brain, and pharmacological CoQ_10_ supplementation or FSP1 activation alleviates ferroptosis-associated injury and improves functional outcomes in models of ischemia–reperfusion and other oxidative stress-related diseases [25]. These findings suggest that the FSP1-CoQ_10_ axis may be particularly important for protecting neuronal membranes and ECs within the NVU under conditions of acute oxidative stress.

A second GPX4-independent defense system is the mitochondrial dihydroorotate dehydrogenase (DHODH)-CoQH_2_ axis. DHODH is a flavin-containing enzyme of the de novo pyrimidine synthesis pathway that is anchored to the outer surface of the inner mitochondrial membrane. During the oxidation of dihydroorotate to orotate, DHODH transfers electrons to the mitochondrial CoQ pool and reduces CoQ_10_ to CoQH_2_ [26]. Mechanistic work has demonstrated that DHODH operates in parallel with mitochondrial GPX4, but independently of cytosolic GPX4 or FSP1, to maintain mitochondrial redox homeostasis; in GPX4-low cells, DHODH becomes the dominant mitochondrial ferroptosis defense, and its inhibition selectively sensitizes such cells to ferroptotic death [26,27]. Given the high energetic demand and mitochondrial density of neurons and ECs, altered DHODH activity may critically influence mitochondrial ferroptosis and thereby contribute to ischemia-induced NVU dysfunction in CVDs [28].

The GCH1-BH4 pathway represents another GPX4-independent defense mechanism that is largely extramitochondrial. GTP cyclohydrolase-1 (GCH1) catalyzes the rate-limiting step in BH4 biosynthesis, converting GTP into dihydroneopterin triphosphate, which is subsequently processed and reduced to BH4. Beyond its classical role as a cofactor for nitric oxide synthases and aromatic amino acid hydroxylases, BH4 functions as a potent lipid radical-trapping antioxidant that directly neutralizes lipid peroxyl radicals and stabilizes polyunsaturated phospholipids against oxidative damage [29]. Studies have identified the GCH1-BH4-phospholipid axis as an alternative ferroptosis defense system that is independent of both GPX4 and CoQH_2_: cells with high GCH1 expression or enhanced BH4 biosynthesis display marked resistance to ferroptosis, whereas genetic or pharmacological inhibition of GCH1 lowers BH4 levels and markedly increases ferroptosis sensitivity [29,30].

Importantly for CVDs, BH4 is also an essential cofactor for endothelial nitric oxide synthase (eNOS). Dysregulation of GCH1 or oxidation of BH4 leads to eNOS uncoupling, reduced nitric oxide bioavailability, and increased superoxide production, which together promote endothelial dysfunction, BBB breakdown, and exacerbation of ischemic brain injury [31,32]. Experimental stroke models have shown that manipulating GCH1/BH4 signaling alters infarct volume, peroxynitrite formation, and BBB permeability, underscoring the dual role of this pathway in both classical oxidative stress and ferroptosis-related lipid peroxidation [31].

Collectively, the FSP1-CoQ_10_-NAD(P)H axis, the mitochondrial DHODH-CoQH_2_ system, and the cytosolic GCH1-BH4 pathway constitute a GPX4-independent CoQ/BH4 antioxidant network. By acting in distinct cellular compartments and cell types within the NVU, these systems complement the System Xc^−^-GSH-GPX4 axis and help determine whether ferroptosis remains controlled or progresses to overt BBB disruption and cerebrovascular injury.

## 3. The Structure, Function and Interaction of BBB

**Figure 2:**
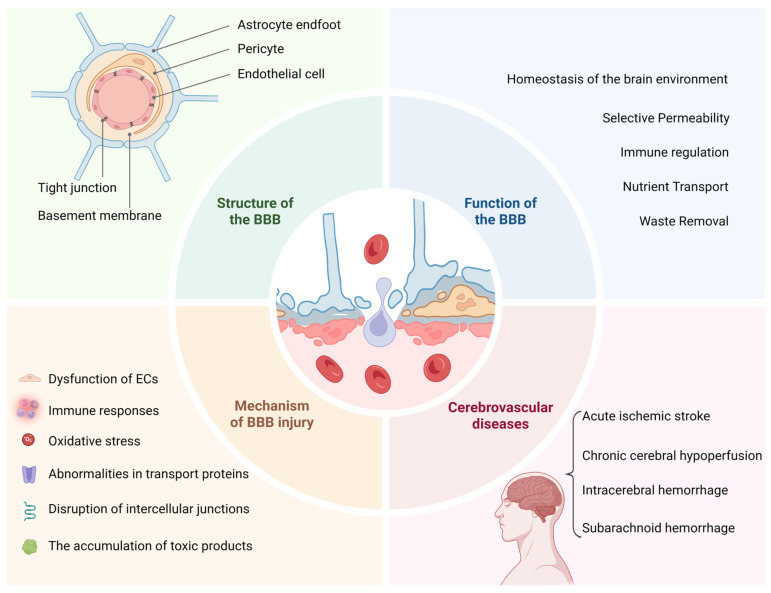
The structure and function of the BBB and the mechanisms associated with BBB damage. Created in BioRender. Guo, J. (2026) https://BioRender.com/up0hx74 (accessed on 8 February 2026).

The BBB lies at the core of the NVU. When the structure or function of the BBB is impaired, harmful blood-borne substances can enter the brain. Inflammatory cells and plasma proteins can also cross. These events lead to tissue injury, white-matter changes, microbleeds, and cognitive impairment. The pathological mechanisms of BBB disruption involve various factors, including dysfunction of ECs, abnormalities in transport proteins, disruption of intercellular junctions, oxidative stress, immune responses, and the accumulation of toxic products. Importantly, almost all CVDs show some degree of BBB disruption (Figure 2). These include ischemic stroke, hemorrhagic stroke, small-vessel disease, cerebral amyloid angiopathy, and vascular cognitive impairment [33,34,35]. Moreover, disease onset, progression, and prognosis are closely linked to the severity of BBB injury. Growing evidence shows that ischemic stroke–induced BBB dysfunction occurs within hours after onset. Cells first swell because ions and water accumulate [36]. The BBB then develops early functional injury. Endothelial transcytosis increases and allows more molecules to cross the barrier. Tight junctions subsequently break down, and paracellular routes open. These changes drive vasogenic edema [37]. Oxidative stress, inflammation, and MMP-2/9 activity amplify this process. Notably, reperfusion or thrombolysis can further aggravate the barrier damage and markedly increase the risk of hemorrhagic transformation. Similarly, recent human imaging studies show that, after acute intracerebral hemorrhage, BBB permeability and neuroinflammation both rise early, although they do not always occur in the same location [38]. Recent imaging studies have also linked BBB disruption to disease progression and poor outcome after subarachnoid hemorrhage (SAH). In a CT perfusion study of 128 patients with aneurysmal SAH, higher Ktrans values, a quantitative marker of BBB permeability, measured within 24 h and again 4–14 days after hemorrhage, were associated with an increased risk of delayed cerebral ischemia, delayed cerebral infarction, and poor 3-month functional outcome [33]. These observations point to one conclusion. We need a deeper understanding of BBB pathology across CVDs.

The major effector cells of the BBB include ECs, pericytes, and astrocytes, with particular emphasis on ECs and the tight junctions between adjacent cells (Figure 2). ECs are intrinsically non-permeable and maintain this barrier property by expressing tight junction proteins—such as claudins, occludin, and junctional adhesion molecules, as well as associated scaffold proteins including onula occludens-1, onula occludens-2, and onula occludens-3 [39,40]. These components form highly sealed tight junction complexes that block paracellular transport, restrict paracellular permeability, and help preserve the homeostatic microenvironment required for neuronal function while preventing the entry of potentially harmful substances. It is important to note that ECs do not function in isolation; instead, they work in close cooperation with other cellular components of the NVU to maintain BBB integrity [41].

Pericytes are located on the abluminal side of ECs and modulate endothelial barrier function through direct physical contact and signaling. Pericytes exert their primary functions by releasing growth and angiogenic factors such as VEGF-A, TGF-β1, and TIMP-3, which promote ECs maturation and protect the basement membrane and tight junctions [42,43,44]. At the same time, pericytes suppress excessive neovascularization and vascular leakage, thereby maintaining vascular homeostasis [45].

Astrocytes enwrap cerebral blood vessels with their end-feet and release a range of factors, including Sonic Hedgehog, Angiopoietin-1, retinoic acid, IGF-1, ApoE, and GDNF, to maintain the integrity of the BBB [46,47]. As a crucial component of the NVU, astrocytes secrete several paracrine factors that primarily target ECs, ultimately influencing the BBB by altering the expression and distribution of tight junction and adherens junction proteins [48].

From a broader view, both neurons and immune cells help regulate the BBB. Microglia are the resident immune cells of the central nervous system, and they can affect neuronal activity and local blood flow when they are resting or activated. Microglia show two phenotypes: the pro-inflammatory M1 type, which weakens BBB function and causes leakage, and the anti-inflammatory M2 type, which supports tissue repair. After brain tissue is injured, microglia become activated very quickly [49]. They release inflammatory factors such as IL-1β and TNF-α, secrete proteases, and increase their phagocytic activity [50]. These signals act on ECs and damage tight junctions. As neuroinflammation increases, BBB permeability also rises. In turn, BBB dysfunction further strengthens the neuroinflammatory response, creating a positive feedback loop. Neurons are central components of the NVU, regulating cerebral blood flow in response to energy demands by releasing vasoactive substances. They also influence NVU function through synaptogenesis, neurotransmission, and maintaining structural integrity. Under pathological conditions, such as in ischemic stroke, hypoxic neuronal damage triggers NVU dysfunction, leading to exacerbated excitotoxicity, oxidative stress, and inflammatory responses. These processes further impair the BBB, worsen cerebral edema, and amplify secondary neuronal injury.

Traditional modes of cell death such as apoptosis, necrosis, and autophagy have been extensively studied in the context of neurological diseases, while ferroptosis, as a new form of cell death, reveals previously unrecognized pathological processes in neurological disorders. Factors that lead to BBB disruption—such as inflammatory responses, oxidative stress, activation of matrix metalloproteinases, and damage to neurons and glial cells—are all closely related to ferroptosis. Evidence suggests that lipid peroxidation in ECs can elevate the levels of MMP-2 and MMP-9, leading to the disruption of tight junction proteins [51]. Lipid peroxidation also induces microglial cells to polarize towards the M1 phenotype, triggering microglia-mediated neuroinflammation. The pro-inflammatory cytokines released by microglia further compromise the BBB, damaging the ECs and tight junction proteins [52]. Meanwhile, the ferroptosis marker ACSL4 has also been shown to be linked to enhanced release of inflammatory factors from microglia during stroke [53]. Additionally, ferroptosis and its associated metabolic pathways are also involved in the dysfunction of other components of the BBB [10,54]. Compared to other cell types, ECs are highly sensitive to hypoxia and oxidative stress, and they show typical features of ferroptosis in many neurological disorders [55]. This may suggest that these ECs have a higher susceptibility to ferroptosis. Taken together, these points suggest that cellular ferroptosis is likely to be one of the important causes of BBB disruption. In CVDs, barrier disruption may provide a pathway for iron leakage, oxidative stress, and inflammation, triggering or exacerbating ferroptosis. In turn, cell damage induced by ferroptosis may further impair barrier integrity. Understanding the relationship between ferroptosis and BBB dysfunction could help identify new therapeutic targets for CVDs.

## 4. Ferroptosis at the BBB in Cerebrovascular Diseases

Accumulating evidence indicates that ferroptosis is intimately involved in BBB breakdown, neuronal death, and neuroinflammation across multiple CVDs, including ischemic and hemorrhagic stroke [51,56]. Iron accumulation and oxidative stress are central to ferroptosis and promote the progression of CVDs by triggering lipid peroxidation and cell death in NVU components [57,58]. Therapeutic strategies that modulate ferroptosis therefore have the potential to stabilize the BBB, attenuate neuroinflammation, and reduce neuronal loss, offering novel avenues for the treatment of brain disorders [59].

### 4.1. Acute Ischemic Stroke

Acute ischemic stroke (AIS), accounting for over 70% of all stroke cases, is fundamentally characterized by an acute obstruction of cerebral arteries by a thrombus or embolus, leading to interrupted cerebral blood flow [60]. This initiates focal cerebral ischemia, hypoxia, and rapidly progressing neurological deficits. AIS triggers a cascade of pathological events, including NVU dysfunction, BBB disruption, neuroinflammation, and reperfusion injury [61]. In animal models such as transient middle cerebral artery occlusion (tMCAO), studies have revealed glutathione depletion, decreased GPX4 activity, and iron deposition within hours post-ischemia [62,63,64]. This is followed by a robust exacerbation of ferroptosis during the reperfusion phase, with brain injury typically peaking 24–72 h after reperfusion [65].

ECs lead to endothelial dysfunction and BBB damage [66]. In rodent AIS models complicated by hyperglycemia, Liu et al. found that activation of the P2RX7 receptor in ECs exacerbates iron overload, ROS generation, and lipid peroxidation, thereby inducing endothelial ferroptosis and consequently aggravating BBB disruption and infarct volume [67]. Chen et al. further demonstrated in hyperglycemic tMCAO rats and cultured ECs that upregulation of the long non-coding RNA Meg3 activates p53, suppressing GPX4 expression and inducing endothelial ferroptosis, which results in more severe endothelial injury and neurological deterioration [68]. Conversely, iron chelation and modulation of mitochondrial iron metabolism have shown protective effects. In mouse ischemia–reperfusion models, administration of iron chelators or overexpression of mitochondrial ferritin to reduce labile iron in ECs and neurons diminished iron deposition, preserved tight junction protein expression, and improved sensorimotor and cognitive functions post-stroke [69,70]. Collectively, these preclinical studies suggest that endothelial ferroptosis is a critical mechanistic link between dysregulated iron homeostasis and BBB compromise.

Astrocytes and microglia are key regulators of BBB integrity and neuroinflammation. Single-cell RNA sequencing of human ischemic stroke tissue revealed a significantly elevated ferroptosis score in astrocytes, indicating an enrichment of ferroptosis-related gene expression [71]. In rodent ischemia–reperfusion models and cultured astrocytes, the damage-associated molecular pattern molecule HMGB1 has been shown to upregulate hepcidin expression in astrocytes via TLR4 and CXCR4 signaling, promoting iron accumulation and ferroptosis, thereby exacerbating ischemic injury [72]. Wu et al. discovered that NDRG2 overexpression in a rat model disrupts astrocytic redox homeostasis through the Wnt/β-catenin pathway, enhancing lipid peroxidation and ferroptosis, which contributes to BBB damage and aggravated neurological deficits [73].

Microglia also undergo ferroptosis under ischemic conditions. In rodent AIS models, microglia in the peri-infarct region display pronounced iron deposition, accumulation of lipid peroxidation products, and upregulation of the ferroptosis marker ACSL4 [74,75]. Duan et al. reported that ischemia–reperfusion induces microglial ferroptosis, which in turn amplifies secondary neuronal damage [76]. To date, multiple animal studies indicate that inhibiting microglial ferroptosis improves outcomes. For instance, Wang et al. engineered “anti-ferroptotic exosomes” that prevented the reduction of M2-type microglia in the acute phase, lowered pro-inflammatory cytokine levels, facilitated BBB repair, and improved neurological function in tMCAO mice [77]. Furthermore, activating antioxidant pathways, such as via Nrf2 or enhancing glutathione metabolism, has also been shown to mitigate microglial ferroptosis, suppress inflammatory injury, and promote neurological recovery [78].

**Figure 3:**
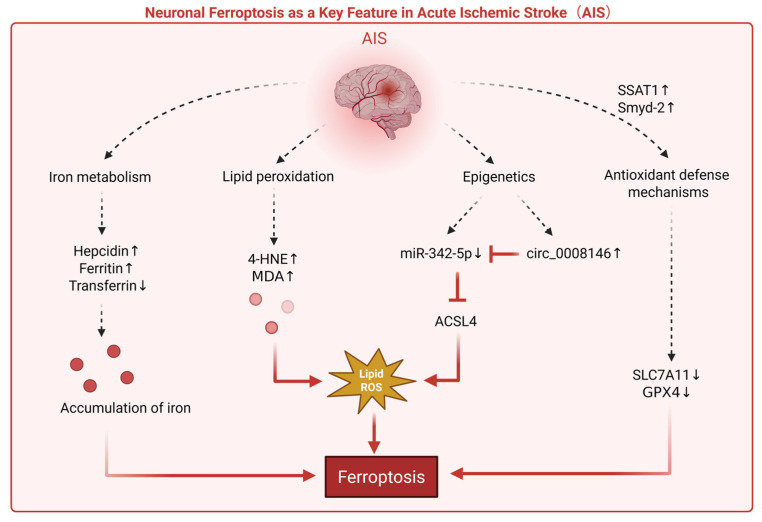
The schematic illustrates the dysregulation of iron metabolism, lipid peroxidation, and epigenetic regulation observed in animal models and in vitro following AIS, as well as the impaired antioxidant defense caused by the overexpression of certain proteins (e.g., SSAT1, Smyd-2). These processes collectively drive neuronal ferroptosis. Abbreviations: AIS, acute ischemic stroke; 4-HNE, 4-hydroxynonenal; MDA, malondialdehyde; ACSL4, acyl-CoA synthetase long-chain family member 4; ROS, reactive oxygen species; SSAT1, spermidine/spermine N1-acetyltransferase 1; Smyd-2, SET and MYND domain-containing protein 2; miR-342-5p, microRNA-342-5p; circ_0008146, circular RNA 0008146; SLC7A11, solute carrier family 7 member 11; GPX4, glutathione peroxidase 4. Created in BioRender. Guo, J. (2026) https://BioRender.com/89xbjzz (accessed on 8 February 2026).

Neuronal ferroptosis is another key feature of AIS (Figure 3). In AIS patients, decreased serum levels of SLC7A11 and GPX4 correlate with stroke severity, suggesting clinical activation of ferroptosis-related pathways [79]. In a mouse tMCAO model, spermidine/spermine N^1^-acetyltransferase 1 (SSAT1) expression is upregulated in the cortical penumbra; complementary in vitro neuronal experiments further suggest that SSAT1 overexpression engages the SSAT1–ALOX15 axis while concomitantly downregulating the antioxidant defense molecules GPX4 and SLC7A11, thereby increasing neuronal susceptibility to ferroptosis and exacerbating oxidative stress [80]. In addition, in ischemia/reperfusion-related models, Smyd-2 overexpression has been reported to inhibit Nrf2 nuclear translocation and is accompanied by reduced expression of Nrf2 downstream targets (such as GPX4 and SLC7A11), similarly promoting ferroptosis and oxidative stress [81]. Beyond the GPX4/SLC7A11 axis, ACSL4—as a key enzyme determining the supply of PUFA-phospholipid substrates—plays a role in ischemic brain injury and inflammatory amplification, suggesting that remodeling of the lipid substrate profile may serve as an important upstream determinant of neuronal ferroptosis susceptibility [62]. Concurrently, epigenetic regulation appears to be involved in this process: miR-342-5p, significantly downregulated in AIS patients, suppresses ACSL4, whereas upregulation of circular RNA 0008146 relieves this suppression by competitively binding to miR-342-5p, thereby exacerbating neuronal ferroptosis and neurological deficits [82].

Based on limited clinical observations and abundant preclinical evidence, ferroptosis signaling in neurons and vascular-related cells is closely associated with infarct size, BBB disruption, and functional outcomes after AIS [67,79]. Rodent ischemia–reperfusion studies indicate that pharmacological inhibition of ferroptosis can attenuate the increase in BBB permeability, reduce infarct volume, and improve neurological deficit scores [83]. In animal models, electroacupuncture/acupuncture and the induction of endogenous anti-ferroptotic transcriptional programs (e.g., selenium-related mechanisms) have also been reported to mitigate ferroptosis-associated injury and improve outcomes [84,85]. From a translational perspective, iron-homeostasis interventions such as deferoxamine have entered early-phase dose and safety evaluation, with a signal of potential benefit observed in subgroups of patients with moderate-to-severe stroke [86]. Meanwhile, effective drug delivery to brain targets remains a major bottleneck, and BBB-targeted delivery strategies are emerging as an active research direction [87]. In summary, significant progress has been made in targeting ferroptosis in AIS, providing potential targets for novel neuroprotective interventions (Table 1).

### 4.2. Chronic Cerebral Hypoperfusion

Chronic cerebral hypoperfusion (CCH) is characterized by a sustained reduction in cerebral blood flow and can lead to cognitive impairment, white matter structural disruption, BBB damage, and neuronal death, and is frequently associated with vascular dementia [88,89]. In animal models of CCH, NVU dysfunction is considered a central driver of white matter injury, acting through compromised BBB integrity, induction of oxidative stress, and activation of inflammatory cascades [61].

Recent studies highlight ferroptosis as an important component of CCH pathology. Fu et al. reported that CCH alters the expression of proteins involved in iron uptake and export in neurons and glial cells, increasing the labile iron pool and thereby enhancing susceptibility to ferroptosis [90]. In a mouse CCH model, Liu et al. further observed iron overload and subsequent cell-death phenotypes in ECs that had internalized myelin debris, suggesting that endothelial injury may contribute to pathological amplification during prolonged hypoperfusion [91]. Meanwhile, Liu et al. reported in a mouse model of CCH that pericyte coverage in the corpus callosum declines early and is accompanied by increased BBB permeability, and that these changes can precede overt white matter injury. This finding suggests that disrupted pericyte–endothelial interactions may represent an initiating event for hypoperfusion-driven BBB breakdown and white matter pathology [92]. However, direct evidence that ECs and pericytes undergo canonical ferroptosis remains limited. Functional validation of GPX4 and ACSL4, as well as rigorous testing with ferroptosis-specific inhibitors in these cell types, is still insufficient. Therefore, further studies in animal models and human tissues are needed to establish stronger causal evidence.

Microglial cell dysfunction during CCH is closely linked to ferroptosis. Adeniyi et al. showed in human brain tissue from older individuals with white matter lesions and patients with vascular dementia that recurrent microvascular ischemia impairs myelination, and the pathological build-up of iron- and lipid-rich myelin debris increases microglial susceptibility to lipid peroxidation and ferroptotic degeneration [93]. These observations provide human evidence for CCH-related white matter pathology and vascular dementia. Consistently, another study reported that myelin debris continuously supplies lipid substrates, driving lipid peroxidation and microglial ferroptosis and reinforcing a vicious cycle of chronic neuroinflammation and BBB dysfunction [94]. In addition, pharmacological inhibition of lipid peroxidation showed microglia-level benefits: methyldopa reduced microglial activation and inflammatory mediator expression in a CCH model [95]. While not validated under stringent ferroptosis criteria, these effects align with alleviated white matter lipid peroxidation.

Beyond glial cells, neuronal ferroptosis is increasingly recognized as a key factor in CCH-induced cognitive dysfunction [88]. According to research by Wang et al., CCH upregulates the expression of miR-30a-5p, which targets and inhibits sirtuin 1, preventing Nrf2 from translocating to the nucleus [96]. This weakens the antioxidant defense and triggers ferroptosis in hippocampal neurons, exacerbating cognitive deficits. Furthermore, multiple interventions—including traditional herbal formulas such as Fo-Shou-San, small-molecule antioxidants such as chlorogenic acid, and metabolic modulators such as dimethyl fumarate—have been reported to activate the Nrf2/HO-1 or Nrf2/GPX4 axis, attenuating iron overload, lipid peroxidation, and neuroinflammation in CCH models and improving spatial memory and executive function [97,98,99]. However, these studies largely rely on pharmacological activation of Nrf2 and infer ferroptosis involvement from reduced ferroptosis-associated markers and behavioral benefits, while rigorous ferroptosis-specific evidence remains limited.

### 4.3. Intracerebral Hemorrhage

Intracerebral hemorrhage (ICH), accounting for 10–20% of strokes, occurs when blood vessels rupture, leading to blood leakage into the brain. It results in high mortality and disability, with poor prognosis largely due to secondary brain injury, including iron-mediated oxidative stress, BBB disruption, inflammation, and edema [100]. After ICH, blood leakage causes iron overload in the perihematomal region, triggering ROS production and lipid peroxidation, key features of ferroptosis [7].

**Figure 4:**
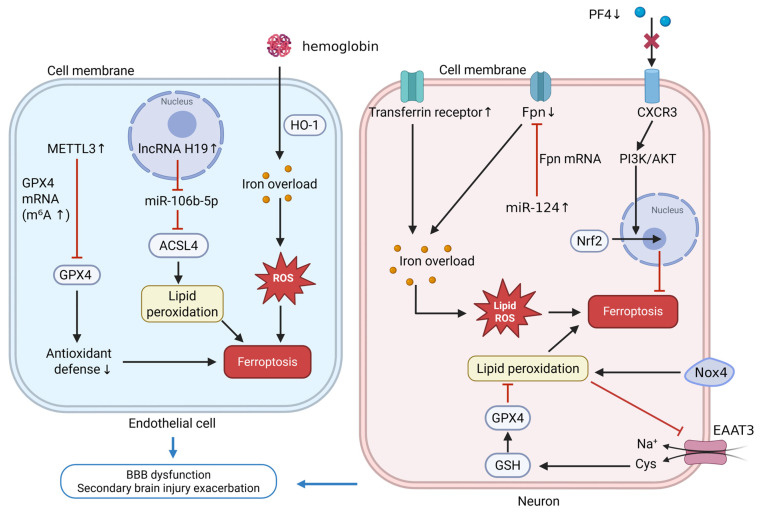
The schematic illustrates how increased iron load and disrupted antioxidant defense following ICH contribute to ferroptosis, BBB disruption, and other pathological outcomes. In endothelial cells (**left**), the lncRNA H19/miR-106b-5p–ACSL4 signaling pathway and METTL3-dependent m6A modification of GPX4 mRNA jointly promote ferroptosis, with overexpressed methyltransferase METTL3 enhancing the m6A modification of GPX4, thereby inhibiting its expression and leading to disrupted antioxidant defense mechanisms. In neurons (**right**), impaired iron efflux (miR-124–ferroportin), NOX4- and transferrin receptor–mediated oxidative stress, EAAT3-dependent cysteine/GSH supply, and the PF4–CXCR3–PI3K/AKT/Nrf2 pathway together determine susceptibility to ferroptosis and the extent of secondary brain edema and neurological deterioration after ICH. The red arrows and symbols in the schematic represent negative regulation or inhibition of downstream signaling. Abbreviations: BBB, blood–brain barrier; HO-1, heme oxygenase-1; METTL3, methyltransferase-like 3; m^6^A, N^6^-methyladenosine; GPX4, glutathione peroxidase 4; mRNA, messenger RNA; lncRNA, long noncoding RNA; H19, long noncoding RNA H19; miR-106b-5p, microRNA-106b-5p; ACSL4, acyl-CoA synthetase long-chain family member 4; ROS, reactive oxygen species; PF4, platelet factor 4; CXCR3, C-X-C chemokine receptor 3; PI3K, phosphoinositide 3-kinase; AKT, protein kinase B; Nrf2, nuclear factor erythroid 2-related factor 2; Fpn, ferroportin; miR-124-1, microRNA-124-1; Nox4, NADPH oxidase 4; EAAT3, excitatory amino acid transporter 3; Cys^−^, cysteine; GSH, glutathione. Created in BioRender. Guo, J. (2026) https://BioRender.com/xr64sb0 (accessed on 8 February 2026).

Research has shown that ferroptosis-induced dysfunction of ECs and pericytes is considered a key factor in BBB damage (Figure 4). Studies have shown that after ICH, the release of iron-containing substances such as hemoglobin and heme leads to the accumulation of Fe^2+^ in ECs and pericytes, glutathione depletion, and an increase in lipid peroxidation products [45,101]. Moreover, in vitro ICH models of ECs, long non-coding RNA H19 is upregulated and functions as a competing endogenous RNA that sequesters miR-106b-5p, thereby releasing ACSL4 from post-transcriptional repression. The resultant ACSL4 overexpression promotes accumulation of PUFA-enriched phospholipids and exacerbates lipid peroxidation, ultimately driving endothelial ferroptosis and BBB dysfunction [102]. Iron chelation or enhancing cellular antioxidant capacity can effectively prevent the related damage. For example, silencing the methyltransferase-like 3 reduces the N^6^-methyladenosine modification of the *GPX4* gene, increasing its mRNA and potentially its protein levels, thereby inhibiting ferroptosis [103]. These findings provide new insights and potential therapeutic strategies for treating ICH.

Ferroptosis is also a critical mechanism of neuronal death following ICH (Figure 4). Multiple studies have elucidated the mechanisms of impaired iron efflux and iron accumulation in neuronal cells. Bao et al. found that miR-124 is upregulated after ICH, inhibiting ferroportin expression, leading to iron accumulation and oxidative stress [104]. Concurrently, Xie et al. further revealed that NOX4 and transferrin receptors are upregulated in the perihematomal tissue of ICH rats, inducing oxidative stress and iron overload. Notably, the study highlighted that GSH depletion in neurons is linked to excitatory amino acid transporter 3 downregulation caused by lipid peroxidation-induced membrane dysfunction, rather than impaired glutamine transport in astrocytes. Excitatory amino acid transporter 3 may thus serve as a potential biomarker for neuronal ferroptosis [105]. Additionally, after ICH, platelet factor 4 levels decrease and this is associated with enhanced ferroptosis. In perihematomal brain tissue of ICH mice and in hemin-treated PC12 neuronal-like cells, platelet factor 4 levels are markedly reduced. Exogenous recombinant platelet factor 4 activates the CXCR3/PI3K/AKT/Nrf2 pathway, upregulates GPX4 and SLC7A11, and suppresses ACSL4 and COX-2, thereby attenuating neuronal ferroptosis and brain edema and improving neurological outcomes [106,107].

Overall, evidence from in vivo and in vitro studies strongly supports ferroptosis as a central hub linking iron toxicity, inflammatory responses, and BBB disruption after ICH.

### 4.4. Subarachnoid Hemorrhage

SAH, primarily caused by ruptured intracranial aneurysms, accounts for 5–10% of all stroke cases. Early brain injury (EBI), occurring within 72 h after SAH, is a major determinant of poor prognosis. EBI involves BBB disruption, cell death, neuroinflammation, and cerebral edema [108]. Clinical and experimental studies consistently show that during this early phase, free iron levels, lipid peroxidation markers and ACSL4 expression are increased, whereas antioxidant capacity is impaired [109,110,111]. Pharmacological inhibition of ferroptosis alleviates BBB disruption and neuronal loss, suggesting that ferroptosis may be an important driver of EBI [112].

In ex vivo preparations of human leptomeningeal arteries, hemoglobin exposure provokes a ferroptosis-like transcriptional profile with ACSL4 upregulation, increased lipid peroxidation and enrichment of ferroptosis pathways, together with disturbance of PI3K–Akt, mitogen-activated protein kinase and vasoregulatory signaling; these findings suggest that iron-dependent injury of endothelial and smooth muscle cells may contribute to vasoconstriction, barrier instability and delayed ischemic deficits after SAH [113]. Consistently, in murine SAH models, ALOX15–driven lipid peroxidation in both microglia and ECs promotes ferroptotic cell death, whereas pharmacological inhibition of ALOX15 with cepharanthine preserves tight-junction proteins, limits BBB leakage [114]. Additional animal and cell-based studies implicate HO-1–mediated iron release and ferritinophagy in astrocytes and microglia as key amplifiers of the labile iron pool [10].

In parallel, glial ferroptosis appears tightly coupled to inflammatory activation and microglial phenotypic polarization. In mouse SAH models with complementary microglial cultures, S100 calcium-binding protein A8-dependent ferritinophagy and autophagy-related ferroptosis are associated with greater neuronal loss and brain edema, whereas S100 calcium-binding protein A8 inhibition reduces microglial ferroptosis and improves functional outcome [115]. In vivo and in vitro microglial studies further show that melatonin suppresses circular RNA ODZ3, upregulates SLC7A11 and GPX4, thereby limiting microglial ferroptosis and shifting cells from a pro-inflammatory M1-like phenotype toward a more reparative state [116]. In similar mouse SAH models and microglial systems, inflammation-targeted single-atom nanozymes combine ROS scavenging with allosteric activation of sirtuin 6, strengthen the xCT–GPX4 antioxidant axis and promote microglial depolarization toward an M2-like phenotype, leading to attenuation of EBI [117]. Combined mouse SAH and neuronal culture experiments also indicate that microglia can transmit ferroptotic stress to neurons: iron-overloaded microglia-derived exosomes are internalized via dynamin-dependent endocytosis, deliver excess iron and complement components, activate a C3/C5–NF-κB cascade and trigger neuronal ferroptosis with aggravated neurological deficits [118]. Together with the endothelial data discussed above, these in vivo and in vitro findings suggest that hemoglobin-driven iron dyshomeostasis not only induces ferroptosis in endothelial and glial cells and disrupts the BBB, but also propagates ferroptotic stress to neurons via iron-rich exosomes and inflammatory signaling within the NVU.

At the neuronal level, studies in mouse and rat SAH models, together with OxyHb- or hemin-treated neuronal cultures and small patient cohorts, have begun to define intracellular programs that govern ferroptotic vulnerability. In prechiasmatic and endovascular perforation SAH models, neurons rapidly downregulate the cystine–GSH–GPX4 defense axis and accumulate labile iron; pharmacological activation of sirtuin 1 in mice restores xCT, GPX4 and FSP1 expression, mitigates cortical and hippocampal ferroptosis and improves EBI indices, whereas sirtuin 1 inhibition aggravates these changes [119]. Consistently, overexpression of the xCT subunit of System Xc^−^ in rats enhances cystine import, replenishes GSH and blocks neuronal ferroptosis, leading to reduced lesion volume and better neurological scores after experimental SAH [120]. Iron metabolism provides a complementary regulatory axis. Zhang et al. showed in a rat EBI model that hepcidin upregulates DMT1 and downregulates the iron exporter ferroportin, shifting neuronal iron trafficking toward intracellular accumulation; inhibition of DMT1 or blockade of hepcidin attenuates iron overload, ferroptosis and functional impairment [121]. More recently, studies in mouse SAH models and hemin-challenged hippocampal neurons have identified additional transcriptional nodes, such as FOXO3a–BAP1 axis that represses SLC7A11 and GPX4 to drive neuronal ferroptosis, and a CoQ_10_ analog that binds and stabilizes FSP1, thereby reinforcing the FSP1–CoQ_10_ system and improving both EBI and cognition in aged SAH mice, with convergent associations between low serum CoQ_10_ and poor outcome in patients [121]

Neuronal metabolism and receptor signaling provide additional layers of control over ferroptosis. In rat SAH models and hemoglobin-treated neurons, the cytosolic enzyme BCAT1 activates the PI3K/AKT/mTOR cascade to maintain GPX4 expression and thereby restrain ferroptosis, whereas BCAT1 knockdown aggravates EBI and lipid peroxidation [122]. An independent stearoyl-CoA desaturase-1 study shows that shifting membrane lipid composition toward a higher proportion of monounsaturated oleic acid attenuates neuronal ferroptosis after SAH, underscoring the lipidome as a key determinant of vulnerability [123]. At the receptor level, acidification of the perihematomal microenvironment activates the proton-sensing GPCR GPR4 on neurons; GPR4 blockade in rat SAH suppresses RhoA/YAP signaling, reduces ferroptosis markers and improves both short- and long-term neurological recovery [124]. In parallel, intranasal administration of recombinant meteorin-like protein in rats engages the C-KIT/AMPK/Nrf2 pathway, upregulates SLC7A11, GPX4 and FSP1, and inhibits neuronal ferroptosis during EBI, whereas genetic or pharmacological disruption of this axis abrogates these protective effects [125]. These in vivo and in vitro studies collectively indicate that neuronal ferroptosis after SAH is not governed by a single linear pathway, but is closely associated with imbalances across multiple processes, including antioxidant defenses, iron transport, lipid metabolism and stress signaling.

## 5. Discussion

CVDs remain one of the leading causes of death and disability worldwide, in large part because current therapies do not adequately address the complex, multicellular pathology of the NVU and the BBB. Although the importance of the BBB in CVDs is widely recognized, therapeutic strategies that specifically target BBB dysfunction are still very limited. In this review, we synthesize recent evidence and propose that ferroptosis—a regulated, iron-dependent form of cell death driven by lipid peroxidation—may represent a common mechanistic axis linking BBB breakdown, neuroinflammation and neuronal loss. This axis appears to operate across both acute and chronic cerebrovascular conditions, including AIS, ICH, SAH and CCH. By considering ECs, pericytes, astrocytes, microglia and neurons as key components of the NVU, we highlight that ferroptosis shows marked cell-type and disease-stage specificity, thereby offering multiple potential entry points for therapeutic intervention in CVDs.

Ferroptosis provides a unifying mechanism that links disturbed iron homeostasis, oxidative stress, inflammation and BBB disruption. Within the NVU, ECs, glia and neurons show distinct but converging vulnerability to iron-driven lipid peroxidation, which promotes junctional breakdown, neuroinflammation and structural injury. Data from ischemic and hemorrhagic stroke as well as CCH models indicate that ferroptosis-related damage closely tracks infarct size, white-matter injury and functional outcome. Taken together, these findings support the view that coordinated ferroptotic injury across NVU cell types destabilizes the BBB.

At the same time, current evidence also exposes several important limitations. Most mechanistic studies rely on rodent models and in vitro systems, which limits their direct extrapolation to human CVDs, and cell-type-specific ferroptosis within the NVU remains poorly defined, particularly in pericytes and astrocytes. In addition, ferroptosis is tightly coupled to other forms of regulated cell death in CVDs: autophagy promotes ferroptosis by degrading ferritin and releasing labile iron, and inhibition of the autophagy receptor NCOA4 can block ferritinophagy-dependent ferroptosis [126]. Key regulatory molecules in necroptosis, such as RIPK1 and RIPK3, are also involved in ferroptosis [65]. These interconnections suggest that targeting ferroptosis alone may not provide stable clinical benefit. Moreover, the BBB remains a major barrier to brain drug delivery: many neuroprotective agents and candidate ferroptosis inhibitors show limited brain penetration, and the spatial heterogeneity of BBB disruption in CVDs further complicates the optimization of dosing, route and timing.

At the same time, current evidence also exposes several important limitations. Most mechanistic studies rely on rodent models and in vitro systems, which limits their direct extrapolation to human CVDs, and cell-type-specific ferroptosis within the NVU remains poorly defined, particularly in pericytes and astrocytes. In addition, ferroptosis is tightly coupled to other forms of regulated cell death in CVDs: autophagy promotes ferroptosis by degrading ferritin and releasing labile iron, and inhibition of the autophagy receptor NCOA4 can block ferritinophagy-dependent ferroptosis, while key mediators of necroptosis such as RIPK1 and RIPK3 also contribute to ferroptotic signaling. These interconnections suggest that targeting ferroptosis alone may not provide stable clinical benefit. Moreover, the BBB remains a major barrier to brain drug delivery: many neuroprotective agents and candidate ferroptosis inhibitors show limited brain penetration, and the spatial heterogeneity of BBB disruption in CVDs further complicates the optimization of dosing, route and timing.

Another prominent and unmet need is the development of stable, clinically feasible biomarkers for ferroptosis and BBB vulnerability. Ongoing work is exploring changes in brain iron burden, lipid peroxidation products, and ferroptosis-related molecules such as GPX4 and SLC7A11 in cerebrospinal fluid or blood as candidate indicators of ferroptotic activity and BBB status. Clinical imaging studies further suggest that BBB permeability maps and quantitative susceptibility mapping-derived measures of brain iron load are associated with outcome after both ischemic and hemorrhagic stroke. However, studies on multimodal biomarker panels remain scarce, and deeper exploration in this area may help to identify patients with a high-ferroptosis phenotype at an early stage and improve risk stratification for vascular cognitive impairment.

Building on these findings, future studies should further clarify the role of ferroptosis across different NVU cell types and disease stages. It will also be important to define systematically how ferroptosis inhibition interacts with current reperfusion and hemorrhage-related therapies (intravenous thrombolysis, mechanical thrombectomy and minimally invasive hematoma evacuation), and to determine whether ferroptosis-targeted approaches can reduce hemorrhagic transformation and edema while extending or stabilizing the therapeutic time window. Beyond acute CVDs, future work should clarify the functional role of ferroptosis and its long-term regulation in chronic cerebrovascular conditions, with particular emphasis on strategies that stabilize the BBB to mitigate ferroptosis-related stress. This framework may be particularly relevant to chronic or mixed cerebrovascular conditions, such as cerebral small vessel disease, cerebral amyloid angiopathy and vascular–degenerative dementias, which may be better suited to therapies centered on long-term BBB stabilization and control of ferroptotic vulnerability.

This review also has limitations that mirror the current state of the field. Most available data are still derived from preclinical studies, many of which rely on indirect indicators of ferroptosis and broad-spectrum antioxidants rather than specific genetic or pharmacological tools. Despite these caveats, integration of existing evidence points to a relatively consistent theme: Ferroptosis is likely to be one of the major driving forces underlying BBB destabilization and NVU failure in CVDs. Thus, viewing CVD pathogenesis through ferroptosis and BBB integrity may facilitate a shift from traditional single-target neuroprotection to integrated, stage-and time window–stratified interventions targeting multiple NVU cell types. If current knowledge gaps and translational barriers can be addressed, and if ferroptosis-related biomarkers and targeted delivery systems are successfully developed and validated, these approaches may ultimately improve long-term neurological outcomes in patients with CVDs.

## Figures and Tables

**Table 1:** Therapeutic strategies targeting ferroptosis in AIS.

Intervention	Type	Experimental Models	Mechanism	Reference
P2RX7 inhibitor; P2RX7 siRNA	Small molecule; genetic	MCAO; OGD/R;	Inhibits P2RX7-driven ferroptotic signaling, preserves GPX4/SLC7A11, reduces lipid peroxidation and endothelial injury.	[67]
Meg3 siRNA	ncRNA knockdown	OGD/R;	Suppresses MEG3–p53 axis, restores GPX4 activity and attenuates endothelial ferroptosis.	[68]
Deferoxamine	Small molecule (iron chelator)	MCAO	Reduces labile iron and iron-driven lipid peroxidation in brain endothelium, thereby attenuating endothelial ferroptosis and limiting post-stroke vasoregression/NVU maladaptive remodeling.	[69]
Mitochondrial ferritin overexpression	Genetic/protein	MCAO/R; OGD/R; ECs	Stabilizes iron homeostasis and mitochondrial redox balance, reducing ferroptosis susceptibility and preserving BBB integrity.	[70]
Tanshinone IIA	Natural product (herbal monomer)	MCAO/R	Improves brain iron handling and redox status, reducing iron-dependent oxidative damage consistent with ferroptosis suppression.	[74]
Minocycline	Repurposed drug	MCAO; OGD/R	Limits HO-1–linked iron release and lipid peroxidation, protecting microglia from ferroptosis and improving injury indices.	[76]
M2pep-ADSC-Exo (engineered exosomes)	Biologic/delivery	MCAO/R	Reduces microglial ferroptosis by regulating the ATF3–SLC7A11 program and reshaping the inflammatory microenvironment.	[77]
A GSNOR inhibitor (compound)	Small molecule	MCAO/R	Activates Nrf2-dependent antioxidant defense and reinforces SLC7A11/GPX4, suppressing microglial ferroptosis.	[78]
SSAT1 knockdown	Genetic	tMCAO/R; primary neurons	Dampens the SSAT1–ALOX15 pro-ferroptotic program, restores GPX4/SLC7A11 defense and reduces neuronal ferroptosis.	[80]
PD146176 (ALOX15 inhibitor)	Small molecule	neuronal ferroptosis assays	Blocks ALOX15-mediated lipid peroxidation, counteracting neuronal ferroptosis.	[80]
Smyd-2 knockdown	Genetic	MCAO/R; OGD/R; HT-22	Relieves repression of Nrf2 signaling, strengthens antioxidant capacity and suppresses ferroptosis.	[81]
ML-385 (Nrf2 inhibitor)	Small molecule (pathway probe)	OGD/R	Confirms Nrf2 dependence by abolishing the anti-ferroptotic protection observed after Smyd-2 suppression.	[81]
Ferrostatin-1	Small molecule	MCAO/R	Inhibits lipid peroxidation and ferroptotic membrane damage; associated with AKT/GSK3β signaling in the cited work.	[83]
Electroacupuncture (EA)	Device-based/non-pharmacological	MCAO/R	Modulates the p53/SLC7A11 axis and reduces ferroptosis-related injury markers.	[84]
Selenium	Nutrient-based pharmacological	MCAO/R	Enhances selenoprotein-dependent anti-ferroptotic capacity, including GPX4-centered defense.	[85]
T-LNPs-ferrostatin-1(BBB-targeted delivery)	Nanomedicine/delivery	MCAO/R	Improves brain exposure of ferrostatin-1 and strengthens anti-ferroptotic neuroprotection.	[87]
circular RNA 0008146 inhibition/miR-342-5p modulation	ncRNA intervention	MCAO/R; OGD/R	Regulates ACSL4-dependent lipid remodeling; miRNA/circRNA perturbation reduces ferroptosis-associated lipid peroxidation and neuronal injury.	[82]

## Data Availability

No new data were created or analyzed in this study. Data sharing is not applicable to this article.

## Abbreviations

The following abbreviations are used in this manuscript:

ACSL4	acyl-CoA synthetase long-chain family member 4
AIS	acute ischemic stroke
ALOX15	arachidonate 15-lipoxygenase
ARE	antioxidant response element
BBB	blood–brain barrier
BH4	tetrahydrobiopterin
CCH	chronic cerebral hypoperfusion
CoQ_10_	coenzyme Q_10_
CoQH_2_	reduced coenzyme Q_10_ (ubiquinol)
CVDs	cerebrovascular diseases
DMT1	divalent metal transporter 1
EBI	early brain injury
ECs	endothelial cells
eNOS	endothelial nitric oxide synthase
FOXO3a	forkhead box O3a
FSP1	ferroptosis suppressor protein 1
GCH1	GTP cyclohydrolase 1
GPX4	glutathione peroxidase 4
GSH	glutathione
HO-1	heme oxygenase-1
ICH	intracerebral hemorrhage
MCAO	middle cerebral artery occlusion
NCOA4	nuclear receptor coactivator 4
NF-κB	nuclear factor kappa B
NOX	NADPH oxidase
Nrf2	nuclear factor erythroid 2-related factor 2
NVU	neurovascular unit
OGD/R	oxygen-glucose deprivation/reperfusion
PF4	platelet factor 4
PUFAs	polyunsaturated fatty acids
ROS	reactive oxygen species
SAH	subarachnoid hemorrhage
SLC7A11	solute carrier family 7 member 11
Smyd-2	SET and MYND domain-containing protein 2
STEAP	six-transmembrane epithelial antigen of the prostate
TfR1	transferrin receptor 1
tMCAO	transient middle cerebral artery occlusion
